# Microcystic serous cystadenoma mimicking pancreatic neuroendocrine neoplasm: report of a resected case with preoperative diagnostic difficulty and review of the literature

**DOI:** 10.1186/s40792-022-01544-0

**Published:** 2022-09-30

**Authors:** Shinichiro Nakamura, Yasuhiro Murata, Katsunori Uchida, Kenichiro Nishikawa, Yusuke Iizawa, Takehiro Fujii, Akihiro Tanemura, Naohisa Kuriyama, Masashi Kishiwada, Shugo Mizuno

**Affiliations:** 1 Department of Hepatobiliary Pancreatic and Transplant Surgery, Mie University School of Medicine, 2-174 Edobashi, Tsu, Mie 514-8507 Japan; 2 Department of Pathology, Mie University School of Medicine, Tsu, Mie Japan; 3 Department of Gastroenterology, Matsusaka Municipal Hospital, Matsusaka, Mie Japan

**Keywords:** Pancreatic serous cystadenoma, SCA, Microcystic serous cystadenoma, Laparoscopic spleen-preserving distal pancreatectomy

## Abstract

**Background:**

Microcystic pancreatic serous cystadenoma (SCA) can be managed without surgery in selected patients. However, the preoperative diagnosis of microcystic SCA remains challenging, and it is potentially misdiagnosed as other pancreatic cystic neoplasms or solid tumors, especially small microcystic SCA.

**Case presentation:**

This was a case of a 27-year-old male patient with microcystic SCA causing difficulty in the differential diagnosis from pancreatic neuroendocrine neoplasm (pNEN). A pancreatic tail mass was incidentally discovered on abdominal ultrasound (US). A contrast-enhanced computed tomography (CT) scan revealed a solid tumor measuring 13 mm with early enhancement in the arterial phase at the pancreatic tail. The tumor showed low intensity on T1-weighted magnetic resonance image, high intensity on T2-weighted image, and a slightly hyperechoic mass on endoscopic US (EUS). EUS-fine needle aspiration (EUS-FNA) did not lead to a definitive diagnosis. The tumor was clinically diagnosed as a pNEN, and a laparoscopic spleen-preserving distal pancreatectomy using the Warshaw technique was performed. The final histopathological diagnosis was microcystic SCA.

**Conclusion:**

Small microcystic SCA is difficult to distinguish from a hypervascular pancreatic tumor such as pNEN on imaging studies, and it is necessary to focus on the tumor echogenicity of EUS to differentiate microcystic SCA from pNEN preoperatively.

## Background

Advancement in imaging techniques has led to an increase in the incidental identification of small pancreatic tumors. The most common pancreatic tumor is ductal adenocarcinoma, and microcystic serous cystadenoma (SCA), a subtype of SCA, is a rare benign epithelial neoplasm [1]. Because microcystic SCA is mostly benign, majority of the patients are only strictly monitored without surgical resection if a definitive diagnosis is established [2]. However, the preoperative diagnosis of microcystic SCA remains challenging, and it is potentially misdiagnosed as other pancreatic cystic neoplasms or solid tumors, especially small microcystic SCA.

Although there might be pitfalls in the clinical diagnosis of microcystic SCA, only a few case reports have focused on investigating the diagnostic difficulty in distinguishing microcystic SCA from other pancreatic neoplasms [3].

Herein, we present a case of a patient with microcystic SCA that was preoperatively diagnosed as pancreatic neuroendocrine neoplasm (pNEN) and underwent laparoscopic spleen-preserving distal pancreatectomy and a literature review including 15 reported cases of microcystic SCA with a difficulty in the preoperative differential diagnosis of pNEN.

## Case presentation

A 27-year-old male patient underwent annual health checkup, and a hyperechoic lesion was incidentally discovered in the tail of the pancreas via abdominal ultrasound. He was referred to our hospital for possible surgery with an inconclusive diagnosis of pNEN. He has no family history of diseases including pancreatic disorders. The blood cell count, biochemistry, and coagulation tests showed absence of abnormal findings. The carcinoembryonic antigen (CEA) level was 4.4 ng/ml, while the carcinoembryonic antigen (CA) 19-9 level was 23.1 U/ml, both of which were within normal range.

**Fig. 1:**
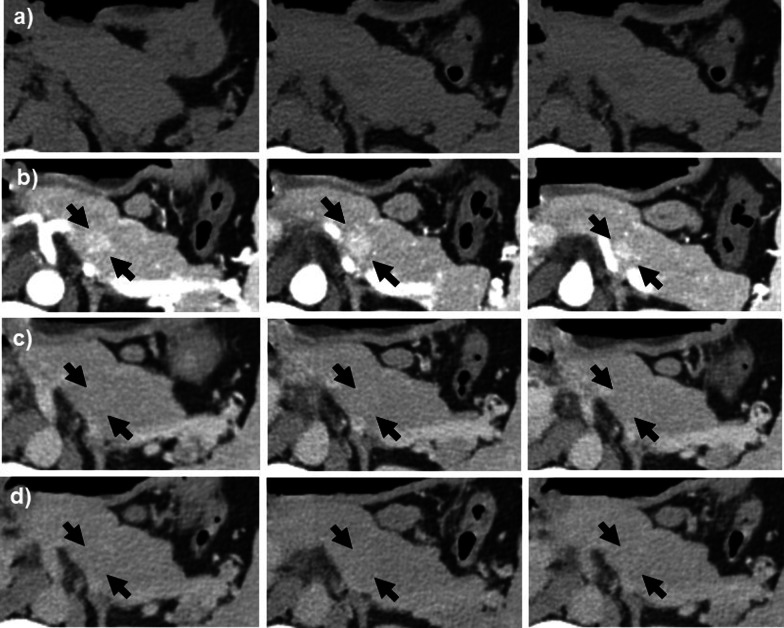
Contrast-enhanced CT of the abdomen. **a** Plain CT, **b** arterial phase, **c** portal phase, and **d** equilibrium phase. A 13-mm-long well-defined mass was found in the tail of the pancreas. The tumor showed slight low density compared with the pancreatic parenchyma on plain CT (**a**) and enhancement in the arterial phase and equal density with the pancreatic parenchyma in the portal and equilibrium phases on dynamic enhanced CT (black arrows in **b**–**d**). Tumor abutment to the splenic artery and vein without encasement was observed. No significantly enlarged regional lymph nodes and distant metastases were noted

**Fig. 2:**
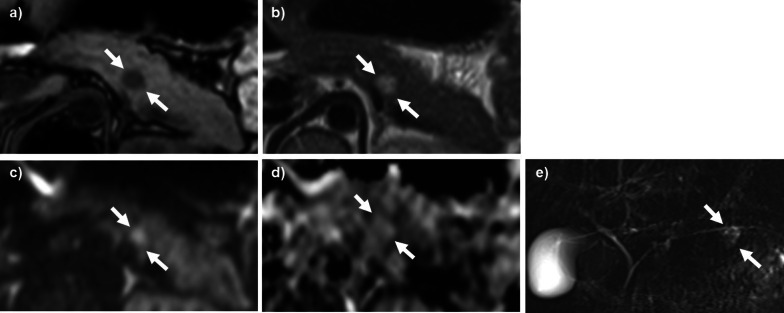
MRI of the abdomen. **a** T1WI, **b** T2WI, **c** DWI, **d** ADC-map, and **e** MRCP. An abdominal MRI revealed a homogeneous mass with low intensity on the T1-weighted image (T1WI), and high intensity on the T2-weighted image (T2WI) and diffusion-weighted image (DWI) and a slightly high intensity on the apparent diffusion coefficient-map (ADC-map) in the pancreatic tail (white arrows in **a**–**d**). A MRCP showed a high-intensity irregular lesion with slight indistinct margins without stenosis and dilatation of the main pancreatic duct in the tail of the pancreas (white arrow in **e**)

**Fig. 3:**
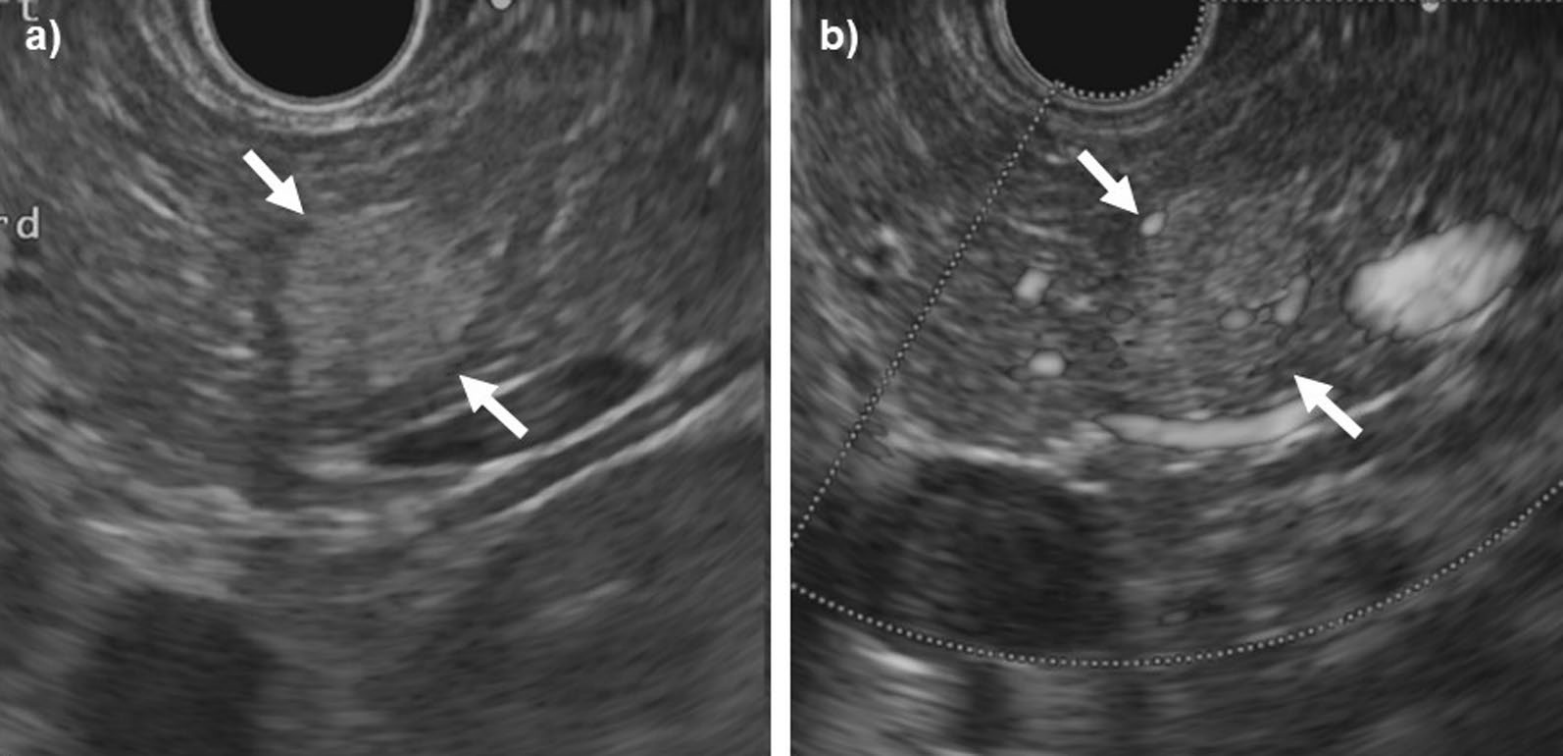
EUS. **a** B mode and **b** color Doppler. EUS revealed a hyperechoic mass with distinct borders and homogeneous interior in the tail of the pancreas (white arrows in **a**). Color flow Doppler imaging showed abundant internal blood flow inside the tumor (white arrows in **b**)

Abdominal computed tomography (CT) examination revealed a well-defined mass measuring 13 mm in the tail of the pancreas. The tumor showed slightly low density compared with the pancreatic parenchyma on plain CT (Fig. 1a), and enhancement in the arterial phase and equal density with the pancreatic parenchyma in the portal phase and equilibrium phase on dynamic enhanced CT (Fig. 1b–d). Tumor abutment to the splenic artery and vein without encasement was observed. The regional lymph nodes were not significantly enlarged, and distant metastases were not noted. Abdominal magnetic resonance imaging (MRI) examination revealed a homogeneous mass with low intensity on T1-weighted image (T1WI) and high intensity on T2-weighted image (T2WI) and diffusion-weighted image (DWI), and a slightly high intensity on the apparent diffusion coefficient-map (ADC-map) in the pancreatic tail (Fig. 2a–d). Magnetic resonance cholangiopancreatography (MRCP) showed a high-intensity irregular lesion with slightly indistinct margins without stenosis and dilatation of the main pancreatic duct in the tail of the pancreas (Fig. 2e). Endoscopic ultrasound (EUS) revealed a slightly hyperechoic mass with distinct borders and homogeneous interior in the tail of the pancreas (Fig. 3a). Color flow Doppler imaging showed abundant internal blood flow inside the tumor (Fig. 3b). The tumor had no visible internal septum and cystic components. The cytopathologic findings by EUS-fine needle aspiration (EUS-FNA) revealed epithelial cells with poor atypia showing a glandular duct structure.

Although a definitive pathological diagnosis could not be obtained, the tumor was preoperatively diagnosed as non-functioning pNEN larger than 10 mm based on the findings of enhanced CT and MRI and EUS-FNA cytology; thus, minimally invasive surgical resection was indicated. Solid-type SCA was one of the possible preoperative differential diagnoses, since the EUS showed a slightly hyperechoic lesion that was a distinctive ultrasonographic feature of a microcystic SCA. As tumor abutment to the splenic artery and vein was observed and spleen preservation was desirable, laparoscopic spleen-preserving distal pancreatectomy was performed following the Warshaw method. The operation was carried out using the 5-port approach. No extrapancreatic invasion of the tumor was observed. In order to preserve the splenic inflow, the distal part of the splenic artery was divided at the proximal site of the root of the left gastroepiploic artery. The operation was considered complete after confirming the absence of changes in the color tone of the spleen. The operation time was 300 min, and the estimated blood loss was 5 ml. The postoperative course was uneventful, and the patient was discharged from our hospital on postoperative day 18.

**Fig. 4:**
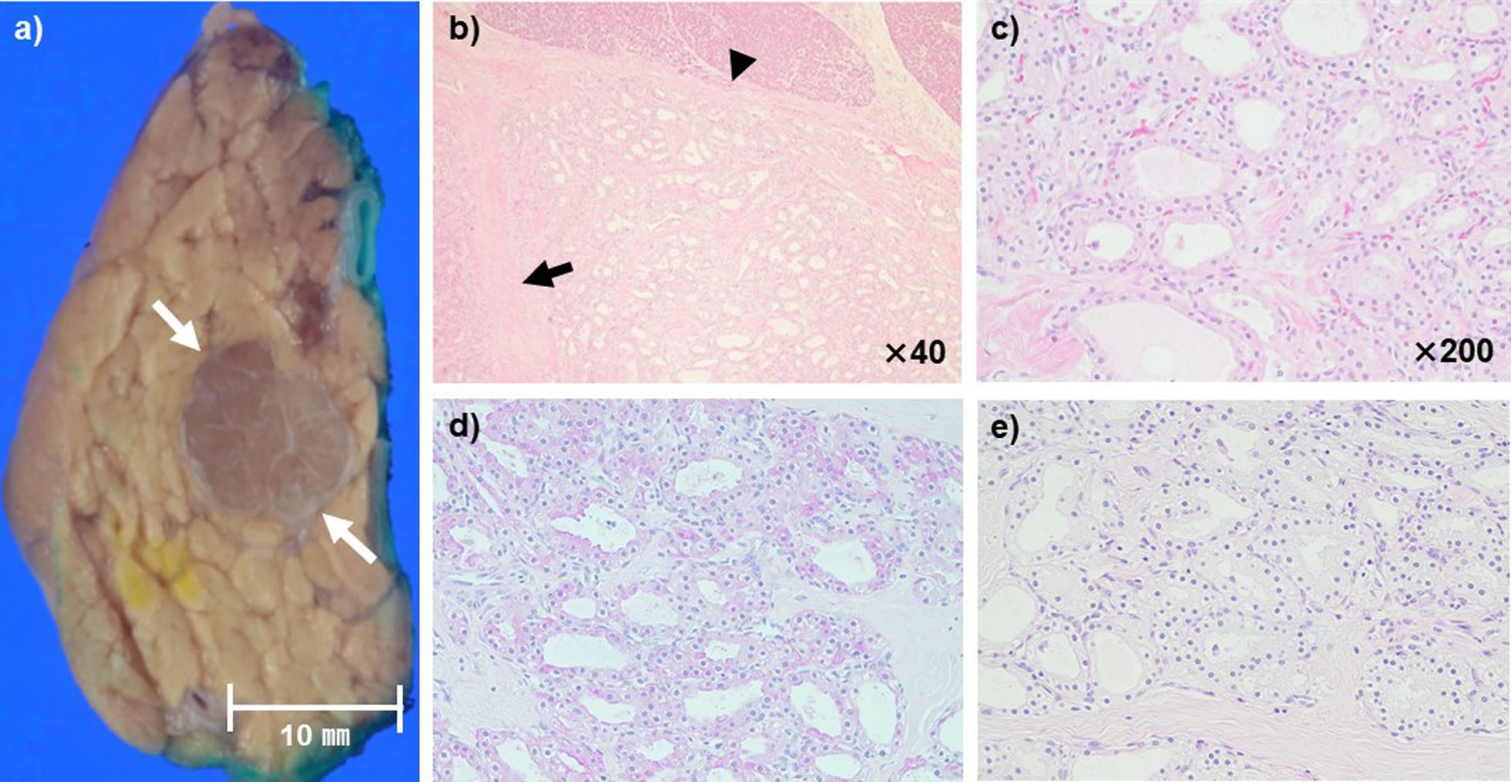
Histopathological findings. **a** Macroscopic image, **b** ×40, **c** ×200, **d** PAS staining, and **e** PAS staining after diastase treatment. Macroscopically, the tumor was a well-defined, round, and solid mass without cystic components measuring 15 mm in size (white arrows in **a**). Microscopically, it appeared as a well-defined lesion with thick fibrous interstitium (arrow in **b**), covered with a membrane (arrowhead in **b**), and composed of epithelial cells having clear vesicles forming microcysts (**c**). The epithelial cells were positive for PAS staining (**d**) before the diastase treatment and negative for PAS staining after the treatment (**e**)

Macroscopically, the tumor was a well-defined, round, and solid mass without cystic components measuring 15 mm (Fig. 4a arrow). Microscopically, it appeared as a well-defined lesion with thick fibrous interstitium (Fig. 4b arrow), covered by a membrane (Fig. 4b arrowhead), and composed of epithelial cells with clear vesicles forming microcysts (Fig. 4c). The epithelial cells were positive for periodic acid Schiff (PAS) staining (Fig. 4d) before the diastase treatment and negative for PAS staining after the treatment (Fig. 4e); the tumor was pathologically diagnosed as microcystic SCA based on the World Health Organization (WHO) classification.

## Discussion

SCA is a relatively rare pancreatic cystic neoplasm that accounts for only 1%–2% of all pancreatic tumors [4]. It frequently occurs in middle-aged or older women and is generally benign. SCA grossly presents as a multifocal cyst containing clear, colorless, serous fluid. Microscopically, it is a glycogen-rich cyst with a monolayer of cuboidal epithelium [5].

Various subclassifications of SCA have been proposed based on the macroscopic and microscopic characteristics. In the 7th edition of the general rules for the study of pancreatic cancer by the Japan Pancreas Society [6], serous cystic neoplasms (SCNs) were pathologically classified into SCA and serous cystadenocarcinoma. Based on the macroscopic classification proposed by Hifumi et al. [7], SCNs are largely classified into Type 1 (microcystic type) and Type 2 (macrocystic type), with subclassification for each type. Type 1 (microcystic type) is subclassified into type 1a (honeycomb type), which consist of small cysts measuring a few millimeters in size and has a honeycomb appearance, and type 1b (solid type), whose cystic spaces cannot be macroscopically identified but can be diagnosed only by microscopic examination. Type 2 (macrocystic type) is subclassified into type 2a (macrocystic dominant type) and type 2b (pure macrocystic type) based on the diameter of the cyst. According to the WHO classification [8], SCNs are classified into five types based on their macroscopic and microscopic characteristics: microcystic SCA, macrocystic (oligocytic) SCA, solid serous adenoma, von Hippel–Lindau-associated serous cyst neoplasm, and mixed serous–neuroendocrine neoplasm. Hifumi’s classification is used to determine the type of SCA based on the macroscopic features, while the WHO classification is used based on the microscopic findings. Therefore, SCA type 1b possibly includes the microcystic SCAs based on the Hifumi’s classification and solid serous adenomas based on the WHO classification. According to the WHO classification of SCA, microcystic SCAs are macroscopically well-circumscribed, slightly bosselated, rounded lesions, sponge like, and composed of numerous tiny cyst (> 0.1–1.0 cm in diameter). Microscopically, the cysts are lined with a single layer of cuboidal to flat epithelial cells with clear cytoplasm, well-defined cytoplasmic borders, and a small round nucleus with dense homogeneous chromatin and an inconspicuous nucleolus [8]. Solid serous adenomas are macroscopically well-circumscribed neoplasms with a solid gross appearance, which show complete absence of cystic change. Microscopically, the subtype is composed of small back-to-back acini with absence or minute central lumina, and cytological features are indistinguishable from those of microcystic SCAs, in the absence of cyst formation [8].

The present case corresponds to type 1b (solid type) based on the macroscopic classification by Hifumi et al., since the imaging findings did not show any cystic morphology but a solid mass with enhancement in the arterial phase on enhanced dynamic CT. Microscopically, the tumor was composed of multiple small cysts, and the pathological diagnosis of microcystic SCA was made based on the WHO classification.

Generally, majority of SCAs are benign neoplasms and are most often managed conservatively with a follow-up [9]. However, surgical resection should be considered in symptomatic tumors with vascular invasion, tumors that are difficult to distinguish from the other malignant pancreatic neoplasms, and tumors showing rapid growth [4]. Several case reports have investigated patients with SCA whose imaging findings closely resemble those of patients with pNEN, and surgical resection was performed due to the difficulty of differentiating SCA from pNEN prior to surgery, as shown in the present case [10, 11]. In addition, the accuracy of EUS-FNA for diagnosing SCA is only 17% [12]; EUS-FNA alone cannot accurately differentiate SCA from other pancreatic neoplasms. Therefore, a preoperative diagnosis of SCA remains a challenge and requires high-level accuracy in interpreting the imaging findings. In the present case, the imaging findings mimic those of pNEN; EUS-FNA did not provide a definitive pathological diagnosis. Hence, it was quite difficult to make a preoperative differential diagnosis between microcystic SCA and pNEN. Hence, we reviewed the literature and compared the imaging findings of the present case with those of a typical case of type 1b (solid type) SCA and a typical case of pNEN (well-differentiated type) (Table 1 [10, 13–20]). In the present case, the plain CT showed a low-density mass, while the enhanced dynamic CT showed an enhancing solid mass with clear borders in the arterial phase and equal density with the pancreatic parenchyma in the portal phase. The large numbers of microcysts in microcystic SCA may appear as a solid mass on contrast-enhanced CT, as shown in the present case [13]. Enhanced dynamic CT is useful in making a differential diagnosis between SCA solid type and pNEN, as the contrast media is washed out from the mass (in a solid-type SCA) during the early phase [14], while pNENs are typically hyperenhancing during the arterial phase and remain mildly hyperattenuating during the portal venous and delayed phases [15]. Since the solid-type SCA contains high water content compared with pNEN, it generally shows a low-density mass on plain CT [14]. MRI typically shows a low-intensity area on T1WI and high-intensity area on T2WI both in solid-type SCA and pNEN [10], meanwhile, pNEN forming a large amount of fibrous tissue shows a low-intensity area on T1WI and T2WI [16]. In the present case, the T1WI and T2WI signals were low and high, respectively, and no cystic components were apparently present in the tumor; this finding suggests that the numerous microcysts could not be detected even on MRI, as the diameter of individual cysts was relatively small. The analysis of signal intensity on the DWI and ADC-map was useful for making a differentiation between solid-appearing SCNs and pNEN [17, 18]. The fluid-rich environments of SCNs likely result in higher ADC values, whereas the decreased extracellular space associated with the dense cellularity in NETs accounts for the lower ADC values [18]. In the present case, the tumor showed a high-intensity area on the DWI and a slightly high-intensity area on the ADC-map with no diffusion restriction, which reflected the presence of water content in the numerous microcysts. Moreover, EUS revealed a hyperechoic mass with distinct borders and homogeneous interior, but no cystic lesion was detected. Color flow Doppler showed abundant internal blood flow inside the tumor. The pNEN typically shows a hypoechoic mass with internal homogeneity reflecting the aggregation of tumor cells [19]. A solid-type SCA shows a hyperechoic mass with clear borders and internal homogeneity on EUS [19]. This SCA type shows a hypoechoic mass on extracorporeal ultrasound and a hyperechoic mass on EUS [20]. The postoperative histopathological findings revealed that the tumor appeared as a well-demarcated mass, and no cyst formation or calcification was grossly observed. Microscopically, epithelial cells proliferated densely with the formation of numerous microcysts and thick fibrous interstitium. Based on the retrospective correlation of imaging findings and results of pathological examinations, the fact that the tumor was pathologically composed of grossly invisible microcysts and thick fibrous tissue strongly indicated that the tumor detected on EUS was a hyperechogenic lesion.

**Table 1:** Comparison of imaging findings between the present case and typical cases of SCA solid type and well-differentiated PNEN

	The present case	SCA solid type	Well-differentiated PNEN
Macroscopic appearance	Solid tumor Well-circumscribed	Solid tumor [17]	Solid tumor [15] Well-circumscribed [15, 17]
Contrasted CT			
Arterial phase	Solid pattern Enhanced	Solid pattern [13] Enhanced [17]	Solid pattern [17] Enhanced [15]
Portal phase	Isoconcentration	Tend to be washed out [14]	Tend to be enhanced [15]
MRI			
T1WI	Low	Low [10]	Low [10, 15, 16]
T2WI	High	High [10]	High [10, 15, 16]
DWI	High	High [17]	High [17]
ADC	Slightly high	High [17, 18]	Low (depending on the degree of malignancy) [17, 18]
EUS	Hyperechoic mass	Hyperechoic mass [20], PEE [19]	Hypoechoic mass [19]

SCA: serous cystadenoma; PNEN: pancreatic neuroendocrine neoplasm; T1WI: T1-weighted image; T2WI: T2-weighted image; DWI: diffusion-weighted image; ADC: apparent diffusion coefficient; EUS: endoscopic ultrasonography; PEE: posterior echo enhancement

We searched PubMed and Medline for clinical series in which surgical resection was performed with a preoperative diagnosis of pNEN and a postoperative pathological diagnosis of SCA was obtained using the keywords “serous cystadenoma” and “pancreatic neuroendocrine neoplasm”. To the best of our knowledge, 15 resected cases of SCA with preoperative diagnosis of pNEN were reported from 2001 to 2021, including the present case (Table 2 [3, 21–32]). In terms of the macroscopic classification of SCA, solid-type SCA [26, 28, 30] and serous solid adenoma [32] were categorized as an SCA solid variant, which Perez-Ordonez B et al. originally described [33]. Four cases that showed few microcysts on histopathological examination with a few microcysts [22–24, 29] were classified as solid serous adenoma based on the WHO classification (Table 2). On the contrary, cases that were diagnosed as “solid variant” or “solid SCA” contained numerous microcysts microscopically and were classified as microcystic SCA based on the WHO classification as in the present case [8].

**Table 2:** Clinical series in which surgical resection was performed with preoperative diagnosis of pNEN and postoperative pathological diagnosis of SCA was obtained

Case no.	Author	Year	Age/sex	Location	Enhanced CT findings	EUS findings	EUS-FNA	Surgical procedure	Postoperative diagnosis
1	Yamamoto et al. [21]	2004	60/M	UP	Hypervascular, uniformly	N/A	N/A	Enucleation	SCA solid variant
2	Yamaguchi et al. [22]	2006	58/F	Pb	Enhanced in arterial phase	N/A	N/A	DP	Solid serous adenoma
3	Reese et al. [23]	2006	66/M	Ph	Hypervascular	N/A	N/A	PD	Solid serous adenoma
4	Sanaka et al. [24]	2007	74/M	Pb	Hypervascular	Hypoechoic	Inconclusive	Enucleation	Solid serous adenoma
5	Yasuda et al. [25]	2011	72/F	Ph	Hypervascular	No cyst	N/A	PD	SCA solid variant
6	Kishida et al. [26]	2014	58/M	Pb	Hypervascular	N/A	N/A	DP	SCA solid variant
7	Sagami et al. [27]	2015	67/M	Ph	Unilocular tumor with wall thickening	Hypoechoic	N/A	PD	Macrocystic type SCN
8	Wu et al. [28]	2015	48/M	Ph	Enhanced in arterial phase	N/A	N/A	PD	SCA solid variant
9		2015	65/F	Pb	Enhanced in arterial phase	N/A	N/A	DP	SCA solid variant
10	Katsourakis et al. [29]	2016	72/F	Pt	N/A	N/A	Inconclusive	DP	Solid serous adenoma
11	Hamid et al. [30]	2017	53/F	Pb	Solid	N/A	N/A	DP	SCA solid variant
12	Yamashita et al. [31]	2018	32/F	Ph	Hypervascular	Hypoechoic	NET	PD	VHL-associated MSNNs
13	Demesmaker et al. [32]	2019	63/M	Ph	Hypervascular	Hypoechoic	Negative	PD	SCA solid variant
14	Nappo et al. [3]	2020	63/M	Pb, Pt	N/A	Hypoechoic	Inconclusive	DP	Microcystic SCA
15	The present case	2021	27/M	Pt	Hypervascular, uniformly	Hyperechoic	Inconclusive	DP	Microcystic SCA

EUS-FNA: endoscopic ultrasound-fine needle aspiration; UP: uncinate process of the pancreas; Ph: pancreatic head; Pb: pancreatic body; Pt: pancreatic tail; DP: distal pancreatectomy; PD: pancreaticoduodenectomy; SCA: serous cystadenoma; SCN: serous cyst neoplasm; VHL-associated MSNNs: Von Hippel–Lindau-associated mixed serous neuroendocrine neoplasms; N/A: not applicable

A review of 15 surgically resected SCAs with preoperative diagnosis of pNEN revealed that most of them were evaluated as a hypervascular tumor with a mass-like appearance on CT scan; none of them, which underwent EUS-FNA, could have led to the definitive pathological diagnosis of SCA. These findings suggest that preoperative differentiation based on EUS-FNA alone can be very difficult and challenging. Preoperative EUS was performed in seven patients including the present case. Of them, five patients presented hypoechoic lesions, which was consistent with a typical well-differentiated pNEN. On the contrary, the present case presented a slightly hyperechoic lesion, which was not a typical ultrasonographic feature of well-differentiated pNEN. Since the tumor was pathologically composed of grossly invisible microcysts and thick fibrous tissue, the slight hyperechoic appearance on EUS was considered to reflect multiple ultrasound reflections from numerous tiny cysts inside the tumor. It was retrospectively considered that a hyperechoic lesion on EUS was a key finding, which might have enabled the preoperative diagnosis of solid-type SCA rather than PNEN. The present case report highlights the usefulness of EUS in differentiating microcystic SCA from pNEN.

## Conclusion

We experienced a case of a patient with microcystic SCA who was preoperatively diagnosed with pNEN and underwent laparoscopic spleen-preserving distal pancreatectomy and reviewed 15 surgically resected SCAs with a preoperative diagnosis of pNEN. Our results indicate that small microcystic SCA is difficult to distinguish from a hypervascular pancreatic tumor such as pNEN on imaging studies, and it is necessary to focus on the tumor echogenicity of EUS to differentiate microcystic SCA from pNEN preoperatively.

## Data Availability

The data sets analyzed during the current study are available from the corresponding author on reasonable request.

## Abbreviations

SCA: Serous cystadenoma
pNEN: Pancreatic neuroendocrine neoplasm
CT: Computed tomography
MRI: Magnetic resonance imaging
EUS: Endoscopic ultrasound
EUS-FNA: Endoscopic ultrasound-fine needle aspiration
CEA: Carcinoembryonic antigen
CA19-9: Carcinoembryonic antigen 19-9
T1WI: T1-weighted image
T2WI: T2-weighted image
DWI: Diffusion-weighted image
ADC-map: Apparent diffusion coefficient-map
MRCP: Magnetic resonance cholangiopancreatography
PAS staining: Periodic acid Schiff staining
WHO: World Health Organization
SCNs: Serous cystic neoplasms
PEE: Posterior echo enhancement
UP: Uncinate process of the pancreas
Ph: Pancreatic head
Pb: Pancreatic body
Pt: Pancreatic tail
DP: Distal pancreatectomy
PD: Pancreaticoduodenectomy
VHL-associated MSNNs: Von Hippel–Lindau-associated mixed serous neuroendocrine neoplasms
N/A: Not applicable
